# "The Hospital" Nursing Mirror

**Published:** 1897-01-30

**Authors:** 


"The Hospital" Nursing Mirror.

Being the Nursing Section of "The Hospital."

[Contributions for this Section of "The Hospital" should be addressed to the Editor, The Hospital, 28 & 29, Southampton Street, Strand, London, W.C., and should have the word "Nursing" plainly written in left-hand top corner of the envelope.]

### News from the Nursing World.

### THE QUEEN'S REIGN.

Princess Henry of Pless is inviting subscriptions from English women married to Germans, and Germans resident in England, towards an offering to the Queen in the shape of a donation to the Queen's Jubilee Nurses. Contributions to be sent, with names and addresses to the Credit Lyonnais, 40, Lombard Street.—At a meeting of the Hereford County Council the question of commemorating the sixtieth year of the Queen's reign came up for discussion, and the Earl of Clarendon (Lord Lieutenant of the county) proposed the establishment of a nurses' fund for providing trained nursing in the rural parishes, or a donation to the Queen's Jubilee Institute, a suggestion which was received with unanimous applause.—At Woking it is proposed to commemorate the "record reign" by raising funds for the building of a new cottage hospital.—At Windsor a suggestion to build a new "Victoria Hospital" seems to be finding much favour amongst the inhabitants.

### PRINCESS HENRY OF BATTENBERG AT VENTNOR.

On January 26th H.R.H. Princess Henry of Battenberg went to Ventnor to the Royal National Hospital for Consumption, the visit being in connection with the proposed building of a new wing in memory of Prince Henry of Battenberg. Her Royal Highness, who was attended by Miss Minnie Cochrane and Lord William Cecil, was received at the hospital by Dr. Coghill, Dr. Whitehead, and several members of the medical staff, and before leaving, after going over the institution, planted two Austrian pine trees as a memento of her visit.

### GUY'S HOSPITAL OUT-NURSING FUND.

A new scheme is about to be started at Guy's Hospital with the object of providing convalescents from the wards and out-patients with skilled nursing in their own homes. In the many cases when a prolonged stay at a convalescent home is not possible after leaving the wards this home nursing will be invaluable, and the diminution in the average stay of patients in hospital in consequence of the subsequent treatment will make much needed room for the reception of more acute and urgent cases. It is estimated that the services of one trained nurse in each district surrounding the hospital would practically augment the accommodation for in-patients by about twenty-five beds at the relatively small cost of £100 per annum for each nurse. A committee of management has been formed, with Mr. Cosmo Bonsor as chairman, Mr. Henry Lafone as hon. treasurer, and Mr. Arthur L. Josephs as hon. secretary. Dr. and Mrs. Perry, Miss Nott-Bower, and Mr. Walter C. C. Pakes are also acting on the committee, and an appeal for funds to support one or more nurses is being issued, towards which donations and annual subscriptions will be gratefully received by the bankers (Union Bank of London, Southwark Branch), hon. treasurer, hon. secretary, or any member of the committee. For the present it is intended to place the nurses in the home of the St. Olave's District Nursing Association, 23, St. James' Road, Bermondsey, which is affiliated to the Queen's Jubilee Institute. This fund is entirely distinct from the Re-endowment and Sustentation Fund.

### MISS WILDMAN'S ACCIDENT.

We are glad to hear that Miss Wildman, whose serious accident on her way out to India we announced the other day, is making satisfactory progress, though it will be some time before she can hope to be about again. Miss Wildman was first taken to the Station Hospital on landing at Gibraltar, but want of room has necessitated her removal to the Colonial Hospital, where she is installed for the present. It may well be imagined that the four days' constant rolling after the catastrophe took place was not the best "environment" for a compound fracture, and of course has militated against quick recovery. The "Demera," the boat by which Miss Wildman was a passenger, had an unlucky voyage altogether, for, after leaving Gibraltar, she got into a terrible gale, the yard-arm broke, the officer of the watch was injured, and one of the soldiers fractured the base of his skull, and had to be landed at Port Said, and taken to the Lady Strangford Memorial Hospital.

### VICTORIA HOME FOR INVALID CHILDREN.

On Saturday last a *café chantant* was organised by Miss F. A. Cooper and Miss Yorke Triscott at the Royal Institute of Painters in Water Colours in aid of the Victoria Home at Margate. Lately two houses facing the sea have been purchased, as being especially suitable for the reception of the little patients, many of whom are drafted from the London hospitals after serious operations, and need all the fresh air and sunshine to be had. The entertainment was got up to clear off a debt of £200 incurred by this outlay. The Duchess of Newcastle is president of the home, and from her, and also from the Duchess of Sutherland, Countess Cadogan, and Lady Ashcombe came a quantity of beautiful flowers for the flower stalls.

### THE BENIN EXPEDITION.

The transformation of the P. and O. steamship "Malacca" into a fitted and equipped hospital ship, supplied with every modern requirement for the comfort and care of the sick and wounded, was carried through in a marvellously short space of time. On Saturday, January 16th, she was actually steaming out of the Thames bound for Antwerp cargo-laden; on that day week she left the Albert Docks for the West Coast of Africa repainted and rearranged from stem to stern, a complete floating hospital with medical and nursing staff on board. Fleet-Surgeon Michael Fitzgerald is in medical charge, accompanied by Staff-Surgeon G. P. Gipps and four naval surgeons, Messrs. J. Grant, G. Macgregor, R. H. Way, and E. Sutton. Three nursing sisters have accompanied the expedition, Miss Isabella Smith, Miss Kate M. Negus, and Miss Eva M'Eogh.

### THE NEW HOSPITAL FOR BELFAST.

The Lady Mayoress of Belfast is to be congratulated on the energy and whole-heartedness with which she has thrown herself into the cause of the proposed new Royal Victoria Hospital. At the ladies' meeting summoned by her she made a very good speech, pointing out the great need for more hospital accommodation in Belfast, and urging her hearers to do all that lay in their power to promote the enterprise. The meeting was well attended, and amongst other speakers were Mrs. Byers, Mrs. Otto Jaffe, and Mrs. Adam Duffin. Some £50,000 have been already promised, and the efforts of the promoters of the scheme are now being devoted to raising this sum to £100,000.

### NURSING AT BURY.

A well-attended meeting was held in the Town Hall at Bury on January 14th to consider the question of starting a branch of the Queen Victoria Jubilee Institute in the borough, at which Miss A. Hughes, late superintendent of the Metropolitan and National Nursing Institution, gave an interesting address. The Mayor, Colonel Walker, was in the chair. A resolution was carried unanimously in favour of the scheme, and a strong committee at once appointed. The Mayor was elected president; Mr. J. Kenyon, M.P., and Mr. O. O. Wrigley, vice-presidents; Mr. E. Milnes, treasurer; and Mrs. Openshaw, secretary.

### TORQUAY NURSE INSTITUTION.

The staff of the Torquay Nurse Institution lately enjoyed a pleasant evening's entertainment, organised for them by the hon. secretary, Dr. Wade. There were tableaux, in which the nurses took part, one of the most successful being a representation of that well-known picture, "The Doctor," and afterwards a concert. Dr. Wade has worked hard in the interests of the institution during the year for which he has held the post of hon. secretary, and with the help of the committee and other friends has succeeded in practically freeing the institution from debt.

### BLACKBURN NURSING ASSOCIATION.

A successful ball on behalf of the Blackburn Nursing Association took place at the Town Hall last week, the entire expense connected with it being defrayed by the generosity of the Mayor and Mayoress. As there was a gathering of nearly four hundred guests, the Nursing Association may expect a very welcome addition to its funds from the sale of tickets.

### STOCKPORT SICK POOR NURSING ASSOCIATION.

The report of the fifth year's work of this association, presented at the recent annual meeting, is satisfactory, if uneventful. There has been an increase in the number of visits paid by Nurses Garner and Tofts, who have been serving the association for two years past, and in whose hands the committee feel the patients' interests are well cared for. The funds from subscribers amounted to rather more than last year, though still the expenditure exceeded the income by about £16. The committee, in gratefully acknowledging gifts of flowers and eggs from various sources, remind the residents in Stockport how great a pleasure such presents give to the sick poor, and ask that more supplies of this nature may be sent to the home for the nurses to distribute.

### WORKHOUSE NURSING AT LEWES.

The impossibility of the present "master and matron" system is well exemplified by recent events at Lewes. Owing to the death of the nurse at the Lewes Workhouse it has been necessary to appoint a successor, and the House Committee interviewed the master and matron and the medical officer on the subject. We read in a local report of a subsequent meeting of the Board of Guardians, that "Dr. Crosskey was in favour of the appointment of a certificated trained nurse, but the master and matron were against it, the latter asserting that certificated nurses were difficult to work with." Mr. Adams, one of the Guardians, might well regret that "the committee had overcome the medical officer in the matter of a certificated nurse," and urge the obvious necessity for a properly-trained head nurse in all workhouse sick wards.

### CONCERT FOR QUEEN'S NURSES.

A concert, held under the auspices of the Rawtenstall District Nursing Society, in affiliation with the Queen Victoria Jubilee Institute for Nurses, was given in the Co-operative Hall on Tuesday evening, January 12th, and proved a great success. There was a large gathering, and the proceedings throughout were highly appreciated; in fact, it was said to be the best concert given in the Co-operative Hall for some time. As a first venture, the authorities of the newly-formed Nursing Association have every reason to be satisfied with the success of this entertainment, and it is to be hoped that the fund will be materially aided thereby.

### NOTTS NURSING ASSOCIATIONS.

An "extraordinary" meeting of the Notts Federation of Nursing Associations was held on January 17th, at the Nottingham General Hospital, presided over by Lady Belper, the particular business on hand being the consideration of affiliating with the Queen's Jubilee Institute, and the question of raising funds. A resolution was passed unanimously recommending such affiliation to the statutory meeting of the federation.

### SHORT ITEMS.

A successful "American sale" was held in the Assembly Rooms at South Molton in aid of the local nursing association.—The Duchess of Fife opens the new out-patient department at the Sussex County Hospital on January 29th.—Mrs. Marcus Dods has been nominated a member of the board of management of the Edinburgh Royal Infirmary. She is the second lady to join the board, Miss Louisa Stevenson having been a member for the past year.—Miss Newport, matron of the Smallwood Hospital, Redditch, organised a pleasant entertainment on January 15th for all patients under 18 who had been in the hospital during the past twelve months.—The nurses of the Glasgow Co-operation for Trained Nurses presented Miss Rough, the Lady Superintendent, with a handsome brass extending floor lamp as a New Year's gift, in token of their appreciation of her untiring efforts for the success of the Co-operation.

### Pharmacy and Dispensing for Nurses.

By C. J. S. THOMPSON.

### I.—DEFINITIONS AND FORMS OF PREPARATIONS USED IN PHARMACY.

As it comes within the province of the nurse in the daily exercise of her calling, not only to handle, but to administer drugs in one form or another, some knowledge of their properties, nomenclature, and methods of preparation cannot fail to be of interest and assistance to her. No complete knowledge of pharmacy, which is essentially a practical art, can be acquired from books alone. We can, therefore, only hope to convey a general idea of this branch of the art of medicine, but one which we trust may lead to a further and practical study of the subject.

In order to understand the peculiarities in nomenclature of the various medicinal agents, it will first be necessary to define the forms and methods of preparation in general use.

In prescriptions, the names of the ingredients are expressed in Latin, and are mainly those included in the British Pharmacopœia, the official list of drugs and chemicals, their compounds and proportions, issued by the General Medical Council, under the authority of the Medical Act. This work fixes the standard for the preparation of the various forms of medicinal agents, and is supposed to include all the reliable remedies required in the treatment of disease. A further object of the pharmacopœia is to cause the various preparations to be made of uniform strength, and to lay down a standard of purity as to the drugs used.

There are, of course, a large number of remedial agents in common use that are not included in the pharmacopœia, and these are classed as unofficial remedies.

A careful study of this work, therefore, is of the utmost importance to the dispenser, who should be familiar with its processes, and know the proportions of the active ingredients in all its preparations.

The contents of the pharmacopœia may be roughly divided into three parts, viz.: (1) The chemical, or inorganic bodies; (2) the vegetable, or organic bodies; (3) the preparations of the above. The nurse should first make herself thoroughly acquainted with the English and Latin names of the chemicals, drugs, and preparations of the pharmacopœia, which are arranged alphabetically in that work, and further, take note of their chief characters and doses.

Taking the British Pharmacopœia as a guide, the first group of preparations we come to are the waters.

The WATERS of pharmacy consist of water holding in solution very small quantities of oils or other volatile principles. Dill, anise, orange flower, carraway, cinnamon, fennel, cherry laurel, peppermint, spearmint, pimento, rose, and elder flower are prepared by distillation; while camphor water is made by maceration, and chloroform water by simply shaking a certain proportion of chloroform and water together.

CATAPLASMS OR POULTICES are soft, moist, local applications employed either for the sake of their moisture and temperature, or on account of certain peculiar active remedies contained in them, such as the poultice of hemlock.

CONFECTIONS are sometimes used as a basis for pill masses only, or in other cases as methods of administering remedies which are but slightly soluble, and require to be given in bulky doses. They are about the consistency of preserves, honey or sugar usually forming the base.

DECOCTIONS are watery solutions of a vegetable and medicinal substance prepared by boiling. With two exceptions, the simple drug, after being prepared, is boiled for ten minutes or longer with a certain proportion of water and then strained.

PLASTERS are usually solid bodies containing some active drug, with wax, soap, and oil as a base. They are melted and spread on leather or other media for application to the body.

ESSENCES are simply solutions of volatile oils in alcohol.

EXTRACTS.—There are fifty extracts, solid and liquid, in the British Pharmacopœia. Some consist of the fresh juice of the drug reduced to the state of solid extract by evaporation. These are commonly termed fresh or green extracts. Others are prepared from the drugs in a dry state by the action of cold or boiling distilled water, by means of which all the soluble matters are dissolved, and the fluid is then evaporated to a proper consistence. The active principles of another class are extracted by means of rectified or diluted spirit, and finally evaporated. The liquid extracts are prepared by a similar process, but the resulting product is not evaporated to dryness.

GLYCERINES are bodies in which glycerine forms the solvent for some more active medicinal substance.

INFUSIONS are simply prepared by allowing a certain proportion of a vegetable drug to infuse in hot or cold water for a period varying from fifteen minutes to two hours, according to the solubility of the active ingredients. There are twenty-eight infusions included in the British Pharmacopœia.

LINIMENTS are fluids of an oily or soapy nature which are employed as external remedies, and either rubbed on or otherwise applied to the body.

SOLUTIONS form an important class in the pharmacopœia. They are mostly watery solutions either of inorganic substances or of certain definite organic principles, such as morphine, strychnine, &c. The solutions of arsenic, atropine, and morphine hydro-chlorate, acetate, and bimeconate, are prepared of the uniform strength of 1 per cent. in water and spirit.

MIXTURES.—The mixtures of the British Pharmacopœia mainly consist of insoluble principles suspended in water by means of gummy or other matters, together with other ingredients.

PILLS.—The pharmacopœia includes twenty-one formulæ for the preparation of pill masses, both simple and compound. These for the most part consist of insoluble principles in powder, combined with some soft substance such as soap, treacle, confection of roses, or glycerine, in order to form them into a suitable consistence for making into pills.

SPIRITS are solutions of volatile oils or other bodies in rectified spirit. The aromatic spirit of ammonia (sal volatile), compound spirit of ether, and the spirit of nitrous ether, are prepared by distillation. Those prepared with volatile oils are made of the uniform strength of one fluid part of the oil to forty-nine parts of rectified spirit.

JUICES consist of the expressed juice of the plant, to which one-third of its volume of rectified spirit has been added to preserve it from decomposition.

SUPPOSITORIES are a form of local application of conical shape, to be administered per rectum.

SYRUPS are liquid preparations in which sugar forms an important ingredient. They are generally used as flavouring agents, or to preserve some active body from undergoing chemical change.

TINCTURES are fluid preparations in which the active principles of the drug are extracted and dissolved in rectified or diluted spirit, or other suitable solvent. Rectified spirit is employed whenever the active portion of the drug is only sparingly soluble in diluted spirit. Proof or diluted spirit is used for making a large number of tinctures in the pharmacopœia, the amount of alcohol present acting as a preservative as well as a solvent, to keep the preparation for a considerable period.

OINTMENTS are semi-solid applications usually employed for smearing over the surface of the body, the base consisting of some fatty matter, such as lard, soft paraffin, or mixtures of wax and oil, &c.

WINES are preparations in which sherry is used as a solvent instead of rectified or proof spirit. They contain much less alcohol than the tinctures, but sufficient to prevent decomposition of their active ingredients.

### Midwifery Papers.

### XIII.—THE NEW-BORN INFANT: GENERAL MANAGEMENT, FEEDING, &c.

GENERAL MANAGEMENT.—As soon after the birth as possible, when the first attention to the mother is complete—having seen that the uterus is properly contracted, and there is no fear of hæmorrhage, that the pad and binder are properly arranged, and soiled articles of all kinds removed from the room—the midwife leaves her patient to rest and turns her attention to the baby. The baby has been awaiting its first bath lying warmly wrapped in flannel in its basket or cradle to which it has been removed from its mother's bed immediately after the cord was tied, and it had indulged in its first good cry. Most babies need their first bath, for they come into the world at times rather thickly coated with the cheesy sticky stuff called vernix caseosa. Having seen that everything she needs is close at hand, the midwife dons her flannel apron and fills the bath with warm water. The temperature of the bath should be 100° when the child is put into it, and for testing this the bath-thermometer should always be used; the hand is a most unreliable guide, and should not be depended on.

Before commencing to bath the child, the midwife should thoroughly cleanse its eyes, and for this a warm solution of perchloride of mercury ($\frac{1}{2000}$) is used and applied on little pieces of cotton wool, instead of a sponge. Every piece of wool used should at once be burnt; it should on no account be dipped into the antiseptic and applied again. The thorough cleansing of the eyes after birth is important, and, if properly carried out, very often does much to prevent the ophthalmia from which so many children, especially of the poorer class, suffer. When the eyes have been cleansed the midwife should wash and dry the child's face and head before soaping it all over and putting it into the bath. Sometimes the vernix caseosa is difficult to get off; it is often especially adherent between the creases of skin on the neck and thighs and over the knee and elbow joints. Too much friction should not be used to get it away, but it should be softened by rubbing on vaseline. After soaping the baby, the midwife should gently place it in the bath, supporting its head and back by her extended left hand, the first finger and thumb gently grasping the upper part of the baby's left arm. The midwife's right hand should be free to sponge the soap and vaseline from the body, and the child should be only allowed to remain in the bath just long enough to remove them, and then it should be lifted on to warm soft towels on the nurse's knee and gently wiped dry. After the bath the cord should be again tied as the first ligature is apt to slip after being wet. If this is neglected and the ligature slips the cord begins to bleed and a serious loss of blood may be the result. It is best to keep the cord as dry as possible; so it should be wrapped in a good many layers of antiseptic gauze or a small pad of absorbent wool, and then turned upwards on the abdomen and the flannel binder applied over it—the binder should not be tightly applied. This treatment should be repeated every day after the bath untill the cord drops off, which it does in a few days. There is generally a little exudation after the cord drops off, while the cicatrix forms, and this can be treated by a little zinc ointment or powder on a small pad of wool or linen or lint. After its bath the baby should be carefully dried, the flexures receiving special attention and dusted over with starch powder. The baby-things should be well-aired and warmed before putting them on. The binders should be secured in place by a few stitches, for pins should never be used in dressing a baby. The baby's clothing should be warm and light, and should allow free movement. The diapers should immediately be changed when soiled, and the buttocks should be cleansed and dried and then powdered. Soiled diapers should be at once removed from the nursery. The nursery should be kept at a warm, even temperature, and care should be taken not to expose the child to draughts, especially when undressing or bathing it.

FEEDING.—Directly the first bath is over the child should be taken to the mother and put to the breast. If the mother is asleep she should not be disturbed, as quiet and repose after the exertion of labour are absolutely necessary. Directly the mother awakes the child should be given to her, for the sooner it is put to the breast the better, as the sucking excites uterine contraction, and the first secretion of milk—the colostrum—is useful to the infant as a natural purgative. For the first two or three days the child should be put to the breast at regular intervals of two hours. The milk, though poor in quality, is generally quite sufficient nourishment for the child. Sometimess, especially if the child is a large one, it seems to require more nourishment than the colostrum affords, and this should then be supplemented by a few teaspoonfuls of warm milk and water (one of milk to two of water) until the full secretion of milk is established. On the fourth day, when the breasts are full and the mother is on a more liberal diet, regular feeding should begin. The regularity of the intervals of feeding is important; irregularity disturbs the quality of the milk, too frequent nursing lessens the water and increases the solids, while too prolonged intervals result in increase of water and decrease of solids. For the first week of a baby's life it should be fed every two hours during the day, at night it should be allowed to sleep as long as possible, but it should be fed when it wakens. After the first week the intervals of feeding should be from two and a half to three hours during the whole period of lactation until it is weaned at the age of ten months. During the later months, especially when the teeth appear, chicken broth, Robb's biscuits, gravy, light milk pudding, stale thin bread and butter may be added to the diet. Milk for the first few years of the child's life is an important item of food, and after it is weaned cow's milk should be daily given in sufficient quantity with its other food. Weaning should take place gradually and cow's milk in daily increasing quantities substituted. It is a good plan to give the heaviest meal of the day about four p.m., as it then comes after and not before the afternoon sleep, and so is more easily digested, for the child is brighter and less inclined to sleep about that time. During the time the child is breast or milk fed its mouth should be carefully washed free of milk after each meal. Milk retained in the mouth is likely to set up a disagreeable condition and perhaps induce "thrush." The mouth can be easily cleansed with a soft wet rag. A healthy child empties the breast in about fifteen minutes. It should then be placed in its cradle to sleep, and should not be allowed to fall asleep while sucking with the nipple in its mouth. Teaching the child to sleep in its cradle from the first saves a great deal of extra trouble, but care should be taken to see that it goes into its cradle with warm feet and a dry napkin. These important items are sometimes overlooked by busy nurses and mothers, and an uncomfortable and crying child is the result. The mother's whole attention should be given to the child while it is taking its meal, to see that the breast is properly emptied and that the child gets its full supply.

ARTIFICIAL FEEDING.—Cases occur occasionally in which it becomes undesirable for a mother to commence or continue suckling her infant, and the best substitute for human milk has to be given to the child. Many patent foods profess to satisfactorily replace human milk, but most of them contain a large amount of starch, and less fat, sugar, albuminoids, and total solids than human milk. The animal-heat producing compounds—fat and sugar—are required in a comparatively large percentage, and in a digestible form, in a young infant's food. Cow's milk is the best substitute, but it has to be modified to suit the child's digestion. It has less sugar and a larger proportion of coagulable albuminoids than human milk. The following preparation has been recommended by Dr. Rotch as most nearly resembling human milk, and has been used with much success. To prepare one pint of food for use in twenty-fours take milk and cream quite fresh and mix as follows:—

| | | | |
|---|---|---|---|
| Milk ... ... ... | 2½ ozs. | Milk sugar, 3⅜ drms. / Water, 3 ozs. | 3 ozs. |
| Cream ... ... | 3½ ozs. | | |
| Water ... ... | 10 ozs. | Lime water... ... | 1 oz. |

Steam for 20 minutes, adding the lime water when slightly warm. Keep the preparation in a cool place, and give the necessary amount warmed at proper feeding time.

The amount given to a child at each meal increases, of course, as the child grows older. The weight of a child is also a guide to feeding—a smaller child requires less food than a larger one. An infant at birth generally weighs from 6 to 8 lbs., and during the first five months of its life has a normal daily increase of ⅔ to 1 oz. Dr. Rotch has prepared a very useful table of general rules for feeding, which I have ventured to insert as a guide:—

| Age. | Intervals of feeding. | No. of feeds in 24 hrs. | Average amount at each feed. | Average amount in 24 hours. |
|---|---|---|---|---|
| 1 week ... ... | 2 hours | 10 | 1 oz. | 10 ozs. |
| 1-6 weeks ... ... | 2½ hours | 8 | 1½ to 2 ozs. | 12 to 16 ozs. |
| 6 to 12 weeks ... | 3 hours | 6 | 3 to 4 ozs. | 18 to 24 ozs. |
| 6 months ... ... | 3 hours | 6 | 6 ozs. | 36 ozs. |
| 10 months ... ... | 3 hours | 5 | 8 ozs. | 40 ozs. |

Great care should be taken in the preparation and administration of the child's food that all vessels used are perfectly clean. The cleanliness of the bottle especially is of the utmost importance. Food infected by a dirty bottle brings with it a train of evils in the shape of diarrhœa, vomiting, and "thrush." The boat-shaped flat bottle, with no tube, and only a nipple, is the best, for it is much more easily kept clean. Two bottles should be kept to be used in rotation. They should always be washed out immediately after use, and when perfectly free from milk should be placed in a pan of clean, cold water. The same rule should be observed in bottle feeding as in breast feeding. The child should be fed on its mother or nurse's knee, and not in the cradle, and the quantity it takes should be noted. The temperature of the child's food should be carefully regulated; it should be warm, not hot or cold.

If the child vomits undigested milk curds, or passes them in its motions, the quantity of milk should be lessened, and a little boiled water be added, at any rate for a day or two, but such an occurrence is almost always the result of sourness of the milk, or dirtiness of the bottle. If care is taken from the first to watch and regulate the quality and quantity of each meal, the distressing vomiting and diarrhœa from which many babies suffer can be avoided. Take the weight as a guide, and remember if the child is small it may be still perfectly healthy, and do not attempt to over-distend its little stomach with an excess of food it cannot digest.

If diarrhœa or vomiting persist, a change of food may be indicated, and condensed milk (1 drachm diluted with water nine times, and 1 drachm of cream added), or peptonised milk should be used for a few days, to rest the stomach and allow it to recover itself. Peptonised and condensed milk are very easily digested, but are not sufficiently nutritious to be continued permanently, and peptonised foods especially should uot be continued for long. If a change of food does not produce the result desired, and the sickness or diarrhœa is not stopped, a doctor should be consulted, for the diarrhœa may be due to causes which require medical treatment.

### Everybody's Opinion.

[Correspondence on all subjects is invited, but we cannot in any way be responsible for the opinions expressed by our correspondents. No communication can be entertained if the name and address of the correspondent is not given, or unless one side of the paper only is written on.]

### AN EIGHT HOURS DAY FOR NURSES.

"A Staff Nurse" writes: Referring to Miss Morrison's objections to an eight hours day for nurses, it is, nevertheless, a fact that, even if it be unadvisable to adopt such a system as this, there should still be more provision made for rest for the hospital nurse. Now, when surgery is becoming more and more a fine art, and that year by year doctors and surgeons require more intelligent care of their cases from us as educated women, we are not exempt from the menial work which public committees exacted from the nurses of former times. We cannot do justice to the important work of our profession, and fulfil the demands of the committee at the same time. To keep one's self surgically clean and be dusting and polishing is incongruous. Our work should not induce such fatigue as to make us impatient with the sick, who are nearly always fretful and fanciful; neither should our leisure be rendered unenjoyable thereby. A course of three years' training in such case is too great a strain; but the difficulty of the situation could be easily met. Nursing is now as much a profession for women as law or medicine is for men. Nurses are now drawn from an educated class; let them pay for their training, if only £10 to £15 for their first year's training, when they are practically of no value to the medical staff. Then let there be more menials to do the polishing of the wards, so that the nurse's attention may be given to her own work, and her leisure be more easily obtainable. Her training would be more complete, and her course of tuition not too great a demand on her physical powers.

### THE MEDICO-PSYCHOLOGICAL ASSOCIATION AND ITS NURSES.

Nurse Jeanie Duddy writes: As a member of the Medico-Psychological Association, may I be allowed space in your paper to express my views on mental nursing—to term it "a degradation" to include mental nurses to any nursing association is utterly ridiculous in our days. There can be no degradation in nursing of any kind. Does Miss S. G. Wingfield consider the nervous system, brain, &c., a separate part of the human body? God created the whole, and as He sees fit to send bodily ailments, so also mental, one is just as much disease as the other; science is certainly not so far advanced in the latter. I have been a mental nurse for the past six years, and not with my eyes shut either. I could mention numbers of most interesting cases, all of which I have most carefully studied, and there is no doubt much might be prevented, habits, &c., by a properly trained mental nurse being called to the case at the commencement, if not altogether a complete cure. Insanity is on the increase; is it not our duty to encourage a better training for mental nurses than heretofore? Asylum nurses of the present day (of course, there are exceptions, as also in hospital trained nurses) are quite on a level with hospital nurses; as anyone experienced in asylum work will understand, and it is not only unjust, but bad form on the hospital nurses' part, to condemn asylum nurses or their work in the present day. As a rule, three ordinary hospital trained nurses cannot manage a mental case half so well as one thoroughly trained mental nurse, who is not only able to undertake the mental part, but the care of the sick in body. What a properly trained mental nurse has to experience during her three years' training is quite as trying as that of a hospital nurse. In many cases her work is very hard, being both mental and bodily strain. A woman who goes in for asylum work is to be admired for her amount of courage—nine people out of ten in this world are frightened to be in the same house with a lunatic, as they term it, much less alone in the same room. There is always risk of a blow from a patient, especially to an inexperienced probationer, and a great deal of tact is required. Therefore we find fewer nurses go in for the training, especially in the case of educated women, who are greatly needed for the care of the insane. The general public opinion of asylum nurses (so-called attendants) seems to be that they must be strongly-built women, who can struggle with a patient if necessary, which shows great ignorance. I am afraid hospital nurses must here be included in the public. I can only add that it is a pity more nurses (educated women) do not take up the work and try and make life easier for the many poor creatures who are confined within asylum walls, many of them left unvisited by their relatives, and in many cases entirely forgotten, and all because the outer world shall not know that a member of their family has been put away in such a place.

"AN OLD NURSE" writes: As an old nurse of nearly a quarter of a century experience, I am grieved to think that an association which has done so much good work as the Medico-Psychological should have made such a blunder as to try and force asylum nurses on to the R.B.N.A.'s register against the desire (even of a few) of its hospital members. In my opinion it is degrading the real mental nurse to do so. Why, instead of all this "fighting and throwing mud" at one another, do not the medical superintendents decide to train their own staff? The asylums do not, like the majority of the hospitals, depend on the charitable to maintain them, and each asylum could afford to pay for an extra medical officer to give lectures, &c.; or I have no doubt several medical officers would only be too glad to do this work for extra remuneration. I am certain if this were done, the medical superintendents would have no difficulty (as to my knowledge many of them have now) in procuring women of a superior class to take up the work, and these would do more towards raising the status of the mental nurse than the R.B.N.A. and Medico-Psychological Council put together can. Mental nursing has made such strides during the last few years, and a better class of women has gone in for the work, that it is grievous it should retrograde through the mistaken policy of forcing its members on a register other than one of their own. For many reasons it is more necessary to have educated and intelligent women as nurses in asylums than in hospitals, because above all things tact, patience, and gentleness as well as firmness are required in nursing the insane, and also the superior class of patients are more in the majority in asylums than in hospitals, for the reason that they cannot be nursed and managed in their own homes, where they could be if they were physically sick. Under all these circumstances, it were well for all asylums to train their own staff on the hospitals' system, and have a register of their own (and if they want it, try and get a royal name tacked on) with the title of B.M.N.A., and not lower the mental nurses by making them a kind of fringe to the R.B.N.A. I am surprised that more mental nurses have not protested against the proceeding. I, for one, consider it an insult to my old branch of the nursing profession to be foisted on to another branch's register. I know that there is a larger number of superior women working in the asylums now, and have no doubt they also must feel it as such. In concluding, allow me to thank you for your championship of the asylum nurse, and, if I am not mistaken, you were the first (some years ago now) to publicly demand the same status to her as to her hospital sister. I have felt grateful to you for this ever since.

### LADY PRIESTLEY'S "PHILIPPIC."

MR. PROSSER JAMES, treasurer of the Hamilton Association for Providing Male Trained Nurses, writes: Allow me to correct an unaccountable error in Lady Priestley's "philippic," as you style her contribution to the *Nineteenth Century*. She points out forcibly enough the necessity of employing male nurses, but confesses that she does not know where to procure them, and gives an erroneous address on the off-chance. It is very discouraging to those who have taken an interest in the matter to find, after years of work and great expense, that a writer in so important a journal should be so utterly at sea on a subject of her own choice. The slightest enquiry would have informed Lady Priestley that the opinion and hope as to male nursing had been anticipated and acted on by one of her own sex. The late Miss Jane Hamilton founded as long ago as 1885 an association for providing male nurses, which has been steadily at work ever since. The expense was defrayed by Miss Hamilton during her life, and she left a legacy to continue the work. The Hamilton Association, as it is named, after its benevolent foundress, is a philanthropic association, not a commercial enterprise, and is presided over by a committee on which the medical profession is strongly represented. For eleven years it has continued to supply trained nurses, and of late some private institutions have been started, apparently with a view of picking up any crumbs of profit. The success of the Hamilton Association is, however, not a pecuniary one. Its object is not to make a profit, but to provide the public with highly trained male nurses and to find these latter employment suitable for their qualifications. It is inexplicable that Lady Priestley should be ignorant of its existence so long after its objects have been commended by the medical and general newspapers, and the years during which its address (57, Park Street) has been regularly advertised in their columns.

### Midwives' Institute and Trained Nurses' Club.

LAST Friday evening the Midwives' Institute and Trained Nurses' Club held its annual business meeting, largely attended in spite of the inclement weather. The President (Miss Wilson) took the chair, and her account of the work and progress of the past year was received with great interest. The Hon. Treasurer then presented the financial statement for 1896. Unfortunately, the popular Secretary (Mrs. M. R. Nichol) was absent through illness, and the Treasurer read her report, which stated that several thousand visitors had used the club-rooms during 1896, that over a hundred new members had been elected, and that the number of letters sent and received were greatly in excess of former years. Towards the close of the meeting several valuable suggestions were made by members regarding future lectures, discussions, &c., to be held at the club, and it is probable that some of them will be carried out very shortly; in fact, it was decided to hold a discussion upon "The Private Nurse and Her Duties" at eight o'clock on Friday, February 19th, when an interesting debate may be expected. When the meeting was over the members were welcomed to a social tea, given by a member of the committee—a very pleasant ending to the evening.

### Appointments.

### MATRONS.

LIVERPOOL CITY HOSPITAL, PARK HILL.—Miss E. Sheldon has been appointed Deputy Matron at this institution. Miss Sheldon was trained at the Workhouse Infirmary, Brownlow Hill, and has since worked at the City Hospital, Park Hill.

CRUMPSALL WORKHOUSE INFIRMARY. — Miss Mary Girdlestone has been appointed Matron and Superintendent of Nurses at this infirmary. Miss Girdlestone received her training at St. Bartholomew's Hospital, afterwards being appointed superintendent of nursing at the Hampstead Workhouse Infirmary, which post she has just resigned.

WORKHOUSE SICK WARDS, HAMPSTEAD. — Miss Amy Frances Lang has been appointed Superintendent of Nursing at Hampstead Workhouse Infirmary. Miss Lang was trained at St. Bartholomew's Hospital, subsequently being appointed superintendent of nursing at the Greek Hospital, Alexandria, She afterwards became acting matron at the Hospital for Paralysis, Regent's Park, and has also had experience in private nursing.

BOROUGH SANATORIUM, KENDAL.—Miss S. M. Jacob has been appointed Matron at this institution. Miss Jacob was trained at Sir Patrick Dun's Hospital, Dublin, where she subsequently was promoted to the post of sister of surgical wards, holding that position for two years. Going from this hospital to the County Meath Infirmary as matron, Miss Jacob remained there for two years, and since June, 1895, has held the appointment of assistant matron at the Borough Fever Hospital, Wolverhampton.

### A Book and its Story.

### THE LIFE OF A GREAT PHYSICIAN.

At his death it was said that "Prophet, philosopher, worker, and saint, they were gathered together in James Young Simpson."* Yet few men have won their spurs in life's battles against more odds than did the subject of this review; and if we may be guided, as, indeed, we may, by his daughter's biography, which has just made its appearance in the world of books, there was little enough in his early surroundings to incite the boy's ambition, unless except it was the heredity, through his mother, of a certain strength of purpose and sunniness of temperament born of French blood. James Simpson's father was the village baker at Bathgate, Linlithgowshire; his ancestry of a remoter period came of a sturdy farmer stock. The future physician, therefore, climbed up the steps of fame from a low rung on the ladder. The climb was not without its attendant difficulties, and lays claim, accordingly, to our unqualified admiration and respect.

It was about the time when the Battle of Waterloo was engrossing British minds, when the magic news had flooded through our land, that young James's school days began. James Simpson's childhood "showed the man as morning shows the dawning." He inherited in full, according to his biographer, the force of character, the independence, the self-reliance of his father's people. His mother had endowed him with her affectionate and hopeful nature, her singularly sympathetic and pleasing manner, and a sweet-toned voice. The boy's career was an earnest of his future. Even from his earliest years his life was a full one, he worked well, and he played well; he had an almost precocious appreciation of time; when in the bakery, and the boy was awaiting customers, his curly head was bent deep over some book. Student life at Edinburgh began for him when he was barely in his teens, and though his future was not yet decided on, he was fired with the determination, whatever should be the line of life ultimately chosen, to be at the "head of his calling." Forty years after, when receiving the freedom of Edinburgh City from the Lord Provost, Sir James Simpson alluded in touching terms to these students days.

The account given of James Simpson's student days before he became famous is, as we have said, the key to his subsequent career.

Into the daily routine of the class-room he infused a vitality, born of keen interest in his subject; to him the dry bones of learning were a thing unknown, and his strong individuality asserted itself against the ordinary acceptance of monotonous formula.

After passing his final examination, the boy—for he was barely more—returned to Bathgate for a while to consider what his next step should be. "It was Professor Thomson who recommended his pupil to take up obstetrics as his special line, and, with that quickness of action which never allowed the grass to grow under his feet, Simpson immediately began to study the subject."

Obstetrics were then treated as low and ignoble among our arts. The youthful student who now took up this branch of physiology as his study, when he died left it a science, numbering amongst its professors many of the most distinguished of our modern physicians. James Simpson's first start in his new career found him lecturer of obstetric medicine in the Extra-Academical School. He was a born teacher, having mastered his subject with a thoroughness, testified by his ultimately being raised to the chair of midwifery. This was in 1840. The rapidity with which event succeeded event at this time of his life was epitomized in a letter—in a friend's letter—to young Simpson. "In May, 1839, you were a lodger, same month a householder; in November, a candidate for the chair; in December, a husband; in February, 1840, a professor; in October, a father."

He was now rapidly gaining popularity, and his practice showed early signs of the extensive proportions into which the physician ultimately developed it. His eyes and ears were constantly on the watch for any new light on science. In three years' time from his professorship he numbered royalty among his patients, and in seven years the Queen appointed him one of Her Majesty's physicians for Scotland. "Flattery from the Queen," the successful physician remarked, "is, perhaps, not common flattery; but I am far less interested in it than in having delivered a woman this week without any pain while inhaling sulphuric ether." The time has now come when the man's genius was concentrated on the long war he fought in the conquering of human pain. His attempts to employ this particular element in obstetric practice were met with oppositions as numerous as they were varied. It was against the dictum of the Holy Scriptures, was one argument among many which was urged against the practice alike by his profession and the world in general. James Simpson was a great physician, great in intellect as in humanity. Nothing daunted by the storm of invective raised against him at this juncture, he continued a brave and steady perseverance in his determination to save his fellow-beings from the unnecessary torment of pain. In his own line of work such a wish was especially applicable; it was given to him to see, what none before him had seen, that there was a saving clause to the pain his women patients were called upon to bear. At this dim hope, for at times so it appeared to him, the great anæsthetist laboured. It was on himself he tested the various drugs which suggested themselves to his mind. Such tests, as may be supposed, were by no means unattended with danger. Chloroform was an unknown quantity before Sir James Simpson immortalised his name through its discovery. Ultimately, with a matured knowledge of its properties, it was tried, cautiously enough at first, on some women patients, the results in the various cases proving successful. The initiatory public test of chloroform was held on November 15th, 1847, within the infirmary where James Simpson had sickened and shrunk from the sight of others suffering. Shortly after this the Queen had the drug administered to her. Her Majesty's example helped to break down the barriers of ignorance and narrow-minded objections still continuing to be urged against the narcotic. It is difficult for us nowadays to throw ourselves back, mentally, into the days preceding anæsthetics—and to be charitable to the scruples evinced by well-meaning persons. Miss Simpson's book is historical in its account of this great period of transition in matters medical. But her father's career is interesting throughout, especially so at the momentous times when the name of Simpson first laid claim to be handed down to posterity with grateful veneration which it is not given to human words to over-estimate.

The scriptural objections raised against chloroform were met by its discoverer in a paper, entitled "The Defence of Anæsthesia," based on the quotation, "Therefore to him that knoweth to do good and doeth it not, to him it is sin." Sir James Simpson's life was not a long one; but it was a full one, and lives are to be judged by what they accomplish more than the span of time they cover. Apart from his discovery, he was a brilliant and a successful practitioner, and when a title came to him late in life he took for his crest the healing rod of Æsculapius, and for his motto, "Victo Dolore." He was offered a last resting-place in the English Abbey wherein lie the great among our dead, but his wife preferred that he should rest in Scottish soil "in the city he had so dearly loved, and which he had chosen as the field of his labours."

* "Sir James Young Simpson." Famous Scots Series. (Edinburgh: Oliphant, Anderson, and Ferrier. 1896.)

### The Book World for Women and Nurses.

A TEXT BOOK OF NURSING FOR HOME AND HOSPITAL USE. By C. WEEKS-SHAW. Edited and revised by WILLIAM J. RADFORD, Resident Medical Officer, Poplar Hospital; with an Introduction by SIR DYCE DUCKWORTH, Physician to St. Bartholomew's Hospital. With numerous illustrations. (Crown 8vo., 388 pages. Edward Arnold, 37, Bedford Street, London.)

When Sir Dyce Duckworth, in his introduction to this admirable volume, modestly claims that "it is one of the best" among several good books on sick nursing, we hardly think he does either it or himself justice. A careful perusal of its pages has convinced us that at present the book has no equal. The aim of the compilers has been to present the various subjects in such a form that the nurse may never be tempted to overstep the boundary line which separates her department from that of the medical man. All the details which have raised modern nursing to the position of a fine art are here elaborately set forth, and we do not ever remember to have seen them so exhaustively dealt with in print. A probationer is as a rule dependent on the staff-nurse or sister of her ward for instruction on such points, and if she should be unfortunately placed as regards either of these, she must pick up information how or when she can. The book before us will be found a real help under such circumstances, for it teaches the reader how to nurse, and to nurse in the most enlightened manner. The opening chapters are devoted to a consideration of the duties and responsibilities of a nurse, the sick-room, and the ward, with excellent regulations as regards their management, ventilation, cleanliness, and good order. Beds and bed-making occupy another important chapter, and the hints with which it closes on the prevention and treatment of bed-sores are among the most valuable in the book. Stress is laid on the necessity for constantly washing the back with soap and water, a point which is only too frequently overlooked in favour of spirit and powder. The application of the latter is, of course, useful as an addition, and we are glad to note a further recommendation, viz., the rubbing in of some simple emollient to keep the skin supple, and then dusting with oxide of zinc and starch in equal parts. Several pages are covered with instructions as to the administration of medicines and enemata, and much valuable information will be found on the subject of leeches, cups, poultices, and fomentations. The latter part of the volume deals with surgical nursing, operations, emergencies, obstetrics, special medical cases, and the diseases of children. Food and its administration forms an appropriate conclusion, and some excellent recipes for fluid nourishment are not the least useful of the subjects embraced. A comprehensive vocabulary completes what may justly be described as a standard work of its kind, and one for which we foresee an extensive circulation. The book is of American origin, and enjoys a wide popularity on the other side of the Atlantic. In adapting it out of the fulness of their knowledge to the needs and requirements of English nurses, we owe Sir Dyce Duckworth and Mr. Radford a debt of gratitude which it is difficult to over-estimate.

### Where to Go.

QUEEN'S HALL, LANGHAM PLACE, W.—A ball in aid of King's College Hospital will be given by Mrs. Wace on February 24th at the Queen's Hall. Tickets may be obtained from Mrs. Wace, King's College, Strand.

EMMANUEL HALL, HARROW ROAD.—A musical cantata will be given at this hall on Monday, February 1st, at eight p.m., in aid of the Temperance Hospital. Tickets, 1s. each, to be obtained from Miss Herbert, 27, Charleville Road, W., or at the hall.

### Minor Appointments.

CUCKFIELD UNION INFIRMARY.—Miss Mary Elizabeth Jenkins has been appointed Head Nurse at this infirmary. Miss Jenkins was trained at the West London Hospital, afterwards holding the post cf superintendent of nurses at the Bethnal Green Infirmary.

### Notes and Queries.

(108) I am wanting to write a paper on the life of Florence Nightingale, and should be much obliged if you would tell me where information about her can be found.—*Irene and Matron.*

There are two lives of Miss Nightingale which might help you, but she has strongly objected to publicity and very little has been said of her in print. The books in question are "Florence Nightingale and Others," by L. Aldridge (Cassell and Co.), 1s.; and "Florence Nightingale, the Wounded Soldiers' Friend," by E. Pollard (Partridge and Co.), 1s. 6d. For accounts of the Crimea consult a file of the *Times* for 1854-55.

*The "Persistent Libels."*

(109) Kindly refer me to the back numbers of THE HOSPITAL in which I shall find recorded (1) "the case of a nurse decamping from a hospital with funds belonging to institution"; (2) "figures for nurses to note and remember"; (3) the case of a nurse who was registered by the Royal British Nurses' Association, although refused her certificate on leaving her hospital; and (4) "the rejection of the Royal British Nurses' Association scheme of registration by the Lords' Committee."—*Impartial.*

You will find full particulars of the cases mentioned in the order given as follows: Case 1. See "Mirror," February 8th, May 24th, June 7th, 1890, pages lxxvii., xxix., xxxviii. Case 2. See "Mirror," May 2nd, 1891, page xxv. Case 3. See THE HOSPITAL, July 2nd, 1892, page 209; and "Mirror," July 2nd, November 12th, November 19th, 1892, pages xcv., xxxix., and xlv.

*South Africa.*

(110) Can trained nurses find employment in Cape Town, and will you tell me what would be the cost of passage and outfit?—*Nurse Mary.*

The Colonial Nursing Association is now acting as a medium of communication between nurses and the Colonies; write to the hon. secretary at the Imperial Institute. Miss Mitchell, Eaton Convalescent Home, Plumpstead, Diep River, S. Africa, will be pleased to give any information to nurses wishing to go out to Africa.

*Home for Aged Women.*

(111) Are there any homes for aged women with brain softening, free or otherwise? Also will you tell me where I could best train in midwifery?—*M. O.*

(1) We do not know of any home especially devoted to the reception of such cases, but doubtless they would be eligible at some of the many homes for incurables either in London or the country. See the list of institutions for chronic and incurable cases in "Hospitals and Charities," p. 848 (Scientific Press, 28 & 29, Southampton Street, Strand, W.C.) (2) Write to the Secretary, Midwives' Institute and Trained Nurses' Club, 12, Buckingham Street, Strand, enclosing stamped envelope for reply.

*Army Nurses.*

(112) Is there any institution where probationers are trained for Red Cross or army nurses?—*M.*

Your query is not very clear. Nurses must have trained for three years in a good general hospital before they are eligible to join the army or navy nursing services. These nursing sisters are given the Royal Red Cross for service to the sick and wounded in war. There is the Red Cross Sisters' Training School for Nurses in Dublin (87, Harcourt Street), but this has nothing to do with the army nursing service. For particulars as to the latter address the Adjutant-General of the Forces, War Office, Pall Mall, S.W.

*Schott Treatment of Heart Disease.*

(113) Will you kindly tell me if I can get a book on this system, with or without illustrations?—*Masseur.*

There is a book on "The Schott Method of the Treatment of Chronic Diseases of the Heart" by Dr. Bezly Thorne, published by Churchill.

*Training at Wandsworth Infirmary.*

(114) Is the Wandsworth Union Infirmary classed as a training school for nurses?—*Inquirer.*

Certainly, and a three years' course of training is given. Read what was said about the Infirmary in the "NURSING SUPPLEMENT," for July 25th, 1896, under the heading "Nursing Arrangements at Clapham," p.cxlv.

*Advice Wanted.*

(115) I am waiting for a vacancy in a fever hospital and would like to study as much as possible during this time. Please recommend me a good book to get.—*Study.*

Dr. Percy Lewis's "Theory and Pratice of Nursing" would probably be the most helpful for you. It is published by the Scientific Press, 28 & 29, Southampton Street, Strand.

*Printed Lists of Expenses.*

(116) Can you tell me where these lists for the use of private nurses are to be obtained? They were advertised in THE HOSPITAL about eighteen months ago, but I forget by whom, and cannot trace them?—*Nurse M.*

We do not quite understand to what lists you can be referring, and remember nothing answering exactly to your description. Please give more details and we will try to trace out what you want.

*Nurses Temperance League.*

(117) Can you tell me how it would be possible to become a member of this league?—*Abstainer.*

This league is only now about to be formed. A meeting in connection with the project is to be held, under the auspices of the W.T.A.U., at Grosvenor House, on February 24th. Write to the Secretary of this latter Society, 4, Ludgate Hill, E.C.

*Spasm of the Heart.*

(118) *Belle*, who asks a question concerning "spasm of the heart," is reminded that no reply can be given in this column unless queries are accompanied by name and address of sender.

